# Comparison of Shock Wave Lithotripsy and Flexible Ureterorenoscopy in the Treatment of 10–20 mm Lower Pole Stone: Prospective Non-randomized Study

**DOI:** 10.7759/cureus.32452

**Published:** 2022-12-12

**Authors:** Cem Kezer

**Affiliations:** 1 Urology, Gunesli Erdem Hospital, Istanbul, TUR

**Keywords:** time between diagnosis and end of treatment, stone size, shock wave lithotripsy, lower-pole stone, flexible ureterorenoscopy

## Abstract

Introduction: The objective of the study is to clarify the efficiency, safety, and limitations of shock wave lithotripsy (SWL) and flexible ureterorenoscopy (f-URS) in the management of lower pole stones (LPS).

Methods: The present study was planned prospectively in a non-randomized manner. Patients who had LPS between 10 and 20 cm in size were enrolled in the study. Patient demographic characteristics, stone-related parameters, complications, and success were noted. Patients who underwent SWL and patients who underwent f-URS were compared according to demographic characteristics, procedure-related parameters, complications, and success rate.

Results: A total of 82 patients matched the study inclusion criteria, with 44 patients treated with SWL and 38 patients treated with f-URS. The time between diagnosis and the end of the treatment was 29.2 days in the SWL group and 15.2 days in the f-URS group (p = 0.001). The success rate was 89.5% with f-URS and 72.7% with SWL (p = 0.036). Receiver operating curve analysis revealed that a stone size larger than 14 mm in the lower pole was significantly associated with SWL failure (area under the curve [AUC]: 0.711, p = 0.033), and a stone size larger than 16 mm was a predictive factor for f-URS failure.

Conclusion: The present study found that f-URS had a significantly higher stone-free rate in the management of 10-20 mm LPS compared to SWL. For the first time, this study showed that the time between diagnosis and the end of treatment was significantly shorter with f-URS. Moreover, LPS larger than 14 mm and 16 mm were predictive factors for SWL and f-URS failure, respectively.

## Introduction

Lower pole stone (LPS) treatment is one of the most controversial issues in daily urology practice. Stone position (lower pole) and size (>1 cm) have been proposed as reasons to opt for more invasive treatments such as flexible ureterorenoscopy (fURS) and percutaneous nephrolithotomy (PNL) to achieve stone-free status. Recently, European Urology Association Urolithiasis Guidelines recommended shock wave lithotripsy (SWL) and endourologic methods for the treatment of 10-20 mm-sized LPS [1]. Even though PNL has satisfactory success rates, the procedure itself can be associated with serious complications like severe bleeding, bowel injury, and pleural injury [2]. Therefore, SWL and f-URS have become more widely used for LPS with 10-20 mm sizes.

There are a number of studies that evaluated the success of treatment modalities for LPS and provided conflicting results. Sener et al. treated 100 patients with f-URS and SWL in a 1:1 ratio, and the authors achieved 92% success with f-URS and 90% success with SWL without any significant difference in success rate [3]. In contrast, Ozgor et al. stated that f-URS had a significantly higher success compared to SWL for the treatment of LPS (89% vs 77.9%, p = 0.029) [4]. In another study, El-Nahas et al. concluded that both procedures did not have significant differences in terms of complications [5].

Although limited numbers of reports compared the stone-free rates and complications of SWL and f-URS for LPS treatment, no study investigated the time between diagnosis and end of treatment or the effect of stone size on SWL and f-URS. The present study aimed to compare the efficiency, safety, and limitations of SWL and f-URS in the management of LPS.

## Materials and methods

The present study was planned in a prospective, non-randomized manner between December 2019 and December 2021. The study was approved by the Ethical Board of Bezmialem University (Meeting Decision No. 2019/178). Patients who had LPS between 10 and 20 cm in size were enrolled in the study. The pros and cons of treatment modalities (SWL and f-URS) were explained in detail to the patients, and the treatment modality was chosen as a result of the patient's decision. All f-URS procedures and SWL sessions were performed by a single urologist. Patients with solitary kidneys and patients with concomitant ureteral stones were excluded. Other exclusion criteria were age <18 years, the presence of a JJ stent or nephrostomy tube, the presence of an infundibulopelvic angle more acute than 30°, multiple stones, and the presence of a non-opaque stone.

Patient demographic characteristics, degree of hydronephrosis, laboratory work (urine analysis, serum creatinine, bleeding profile), and previous stone treatment were noted. Moreover, stone characteristics, including stone side, stone size, and Hounsfield Unit (HU), were evaluated by computed tomography urography. Also, analgesia requirements, the time between diagnosis and end of treatment (days), complications, and stone-free status were recorded. The stone size was accepted as the longest stone length in all axes, and the infundibulopelvic angle was measured between the infundibular and ureteropelvic axes in the computed tomography images. The duration of treatment for SWL patients was calculated as the time from the diagnosis to the end of the treatment (stone-free status or termination of the procedure due to rest calculus), and for f-URS patients, the time from the diagnosis to the day of operation.

SWL and f-URS technique

All f-URS operations were performed under general anesthesia. A safety guide wire was placed into the ureter using cystoscopy, and an 8-F ureterorenoscope (Karl-Storz, Tuttlingen, Germany) was inserted into the ureter to detect the presence of ureteral pathology and to facilitate the placement of a ureteral access sheath (UAS) (11/13F). If the UAS insertion failed, we used f-URS without the UAS. A 7.5-F flexible ureterorenoscope (Storz FLEX-X 2, Tuttlingen, Germany) with a Holmium laser was performed for stone fragmentation. Fragmentation was started with a dusting (1.5 J and 10 Hz), and after the fragments were reduced in size, popcorning (0.8 J and 15 Hz) was applied. If possible, stones were relocated in the upper and middle calyces for easier processing. JJ stent was placed in all patients at the end of the operation. A nitinol basket was used for the extraction of stone fragments. SWL treatment was performed using the Pck Calculith Gold Basic Electromagnetic (Pck Medical, Ankara, Turkey). The procedures were performed under the guidance of ultrasonography or fluoroscopy. Before each SWL session, intramuscular analgesia was administered, and SWL was initiated at a frequency of 60 shocks/minute, with the frequency rising to a maximum of 90 depending on patient tolerance. In each session, 2000 to 2500 shocks were delivered. In the presence of rest calculi after the procedure, the sessions were continued, and a maximum of three sessions of the procedure were applied.

The patient was considered stone-free if no stone fragment on kidney-ureter-bladder graphy and urinary ultrasonography was detected six weeks after the procedure. Patients were compared according to preoperative data, procedure-related parameters, complications, and stone-free rates. In addition, the effect of stone size on stone-free rates of SWL and f-URS was analysed.

Statistical analysis

The Statistical Package for the Social Sciences, version 25 (SPSS IBM Corp., Armonk, NY, USA) program was used for statistical analysis. The variable distribution was evaluated with the Shapiro-Wilk test and Q-Q plots. Quantitative data are presented as mean ± standard deviations. Categorical variables were analyzed by the χ2 test. Receiver operating characteristic (ROC) curve analysis was done to define the cut-off value and the area under the curve (AUC), with the 95% CI and p-value ≤ 0.05 considered statistically significant results.

## Results

A total of 82 patients matched the study inclusion criteria, with 44 patients treated with SWL and 38 patients treated with f-URS. Age, male/female ratio, body mass index (BMI), history of previous renal stone surgery, HU of stones, and serum creatinine level were comparable between groups (p = 0.990, p = 0.618, p = 0.333, p = 0.673, 0.498, and p = 0.993, respectively). The mean stone size was 13.5 mm in the SWL group and 14.1 mm in the f-URS group (p = 0.214). The number of treatment sessions was significantly higher in the SWL group (Table 1).

**Table 1:** Comparison of demographic data between groups *Mean ± standard deviation, SWL: shock wave lithotripsy, f-URS: flexible ureterorenoscopy, BMI: body mass index

	SWL (n=44)	f-URS (n=38)	P value
Age (years)*	41.6±14.7	41.5±11.6	0.990
Sex (male/female)			0.618
Male	29 (65.9%)	27 (71.1%)	
Female	15 (34.1%)	11 (28.9%)	
BMI (kg/m^2^)*	25.7±3.7	26.4±3.4	0.333
Previous stone surgery	11 (25.0%)	8 (21.0%)	0.673
Presence of hydronephrosis			0.967
Absent	17 (38.6%)	15 (39.5%)	
Grade 1	23 (52.3%)	19 (50.0%)	
Grade 2	4 (9.1%)	4 (10.5%)	
Hounsfield unit	789.7±284.2	829.5±237.6	0.498
Stone size (mm)*	13.5±2.4	14.1±2.1	0.214
Serum creatinine (mMol/L)*	88.8±22.3	88.9±24.5	0.993
Side			0.922
Right	25 (56.8%)	22 (57.9%)	
Left	19 (43.2%)	16 (32.1%)	
Number of treatment sessions*	2.2±0.7	1.0	0.001

Analgesia requirements did not significantly differ between groups (p = 0.537). The time between diagnosis and the end of the treatment was 29.2 days in the SWL group and 15.2 days in the f-URS group, and the difference was significantly shorter in favour of f-URS (p = 0.001). Four patients in the SWL group and three patients in the f-URS group experienced complications. Sepsis developed in one patient in the f-URS group, and the patient was discharged after IV antibiotherapy and hydration. The stone-free rate was 89.5% with f-URS and 72.7% with SWL, and stone-free status was significantly higher with f-URS (p = 0.036) (Table 2).

**Table 2:** Comparison of post-procedure data between groups *Mean ± standard deviation, SWL: shock wave lithotripsy, URS: ureteroscopy, NA: not applicable.

	SWL (n=44)	f-URS (n=38)	P-value
Analgesia requirements	25 (56.8%)	19 (50.0%)	0.537
Stone free status	32 (72.7%)	34 (89.5%)	0.036
Time between diagnosis and end of treatment (days)*	29.2±7.3	15.2±4.9	0.001
Complications	4 (9.1%)	3 (7.9%)	0.847
Clavian-Dindo grades 1-2	4 (9.1%)	2 (5.3%)	NA
Clavian-Dindo grades 3-5	0 (0%)	1 (2.6%)	NA

**Figure 1:**
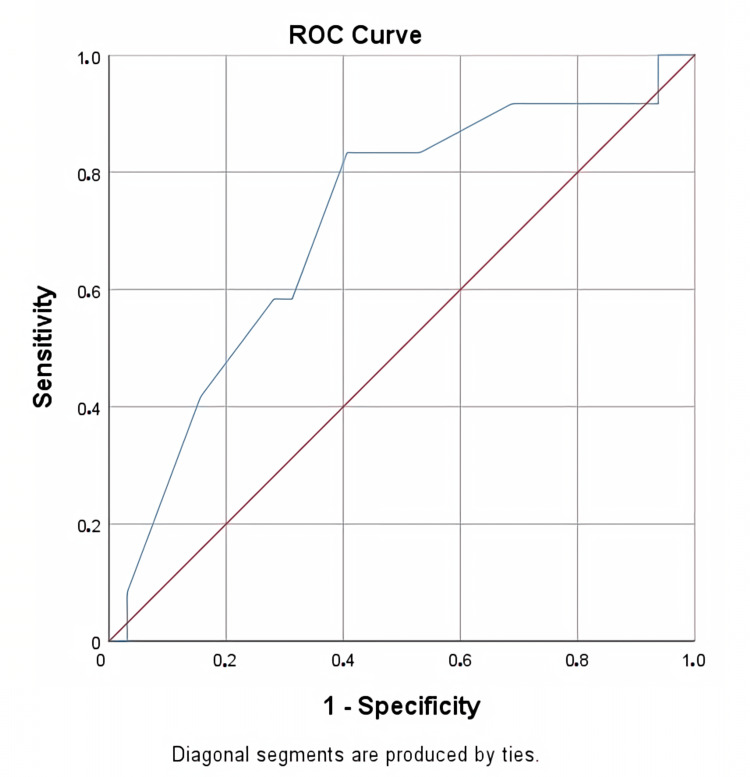
Receiver operating curve analysis of the effect of kidney stone size on shock wave lithotripsy

**Figure 2:**
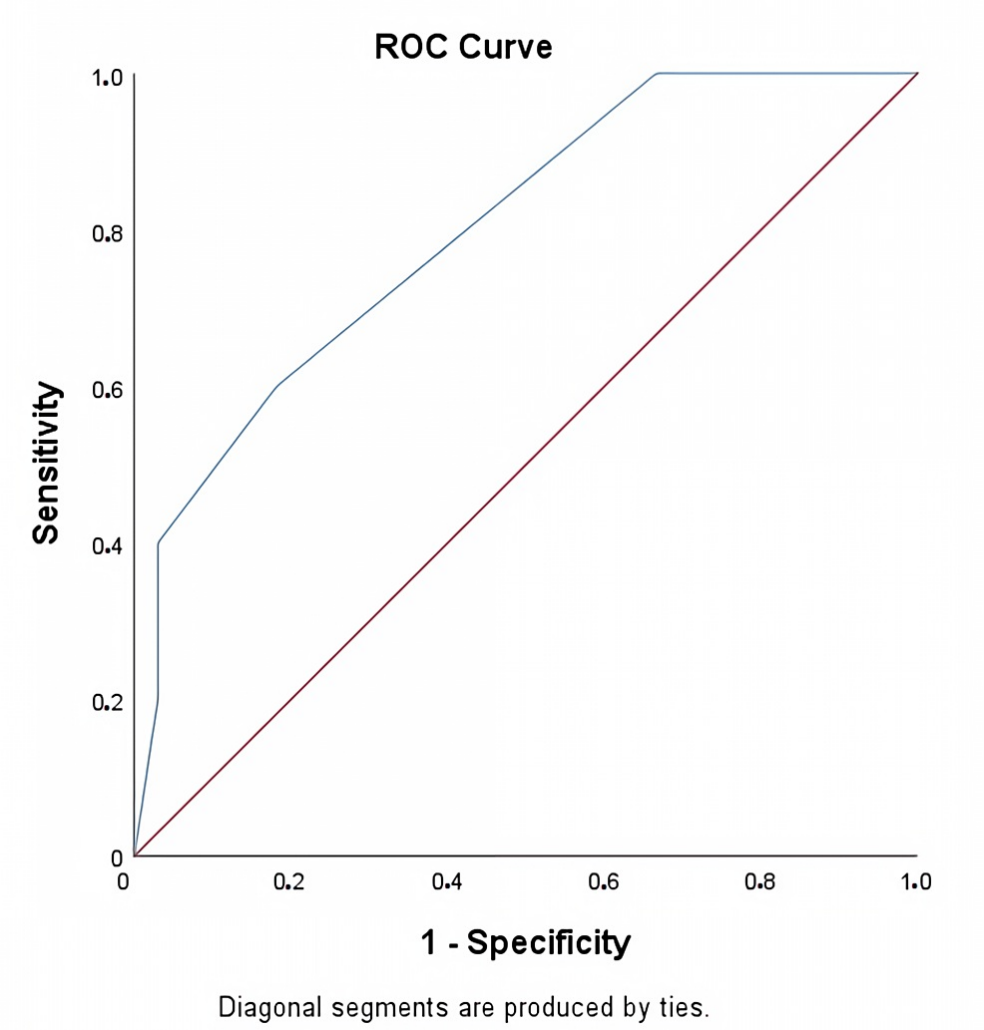
Receiver operating curve analysis of the effect of kidney stone size on flexible ureterorenoscopy

Receiver operating curve analysis revealed that a stone size larger than 14 mm in the lower pole was significantly associated with the stone-free status of SWL (AUC; 0.711, p= 0.033) (Figure 1). Additionally, the receiver operating curve showed that a stone size larger than 16 mm was a predictive factor for the stone-free status of f-URS (AUC; 0.800, p = 0.023) (Figure 2). The effect of stone size on SWL and f-URS stone-free status is summarised in Table 3.

**Table 3:** Receiver operating curve analysis of the effect of kidney stone size on stone-free status of SWL and f-URS AUC: area under the curve, SWL: shock wave lithotripsy, URS: ureteroscopy

	AUC	Cut-off point (mm)	Sensitivity	Specificity	P-value
SWL	0.711	>14	0.583	0.688	0.033
f-URS	0.800	>16	0.628	0.818	0.023

## Discussion

The management of LPS is one of the most interesting topics in urology, and discussions continue about the best treatment option. There are a limited number of comparative studies on the treatment of LPS with SWL and f-URS [6-8]. In this prospective study, we found a significantly higher stone-free rate following f-URS compared to SWL with similar complication rates. In addition, the present study showed for the first time that the time between diagnosis and the end of treatment was significantly shorter with f-URS. Moreover, stone sizes >14 mm and >16 mm were associated with failures of SWL and f-URS, respectively.

Achieving stone-free status with minimal complications is the main purpose of treatment modalities. For the management of LPS with a 10-20 mm size, Zhang et al. achieved a significantly higher success rate with f-URS compared to SWL, with similar complication rates [9]. In another study, Mi et al. reviewed the studies about SWL and f-URS, they claimed that f-URS increased the stone-free status 2.35 times for LPS with 10-20 mm size in comparison with SWL. Additionally, Mi et al. stated that f-URS did not increase the complication rate compared to SWL [10]. In accordance with the aforementioned studies, f-URS achieved a higher stone-free status without increasing complications in the present study.

Renal stone size is an important factor for treatment choice as well as for predicting procedure success. Azal Neto et al. analysed data from 1902 patients who underwent SWL, and the authors found a 73.8% success rate for renal stones <1 cm, a 70.4% success rate for renal stones with 1-1.5 cm, and a 56.2% success rate for renal stones >1.5 cm [11]. Similarly, Tsai et al. stated there was a decrease in SWL success for LPS with increasing stone size [8]. Karagoz et al. obtained 14.28% and 75% success rates for LPS <20 mm and >20 mm, respectively (p = 0.001) [12]. Moreover, Akman et al. found that increasing stone volume was a risk factor for additional procedures following f-URS [13]. However, none of the aforementioned studies gave a cut-off stone size for procedure failure. In this study, stone sizes >14 mm and >16 mm were predictive factors for stone-free rates of SWL and f-URS.

Delayed treatment of any disease can be associated with disease progression, increments in mortality, and increases in healthcare budgets. McKinley et al. stated that delays in cardiac pathologies (acute coronary syndrome, myocardial infarction) decreased the benefits of cardiac treatment [14]. In another study, Waldstein and Katzel stated that delayed diagnosis of hypertension was a predictive factor for cognitive brain functions [15]. However, no study evaluated the time between diagnosis and end of treatment in the management of LPS. For the first time, the present study showed that this period was significantly shorter for f-URS. Future prospective randomised studies will show the impact of shortened diagnosis-treatment durations in clinical practice.

Although it was a prospective study, the small patient number could be accepted as a limitation. Second, this study included patients’ data from a single institution. However, this condition prevents possible bias about a surgeon's skills and experience. In addition, this study only analysed the short-term outcomes of both treatment modalities; the long-term outcomes of these procedures could be evaluated in further studies. The patients in the SWL group were evaluated for all sessions, not separate sessions. Lastly, the costs of procedures were not compared in the present study, which could be the subject of another study.

## Conclusions

The present study found that f-URS had a significantly higher stone-free rate in the management of 10-20 mm LPS compared to SWL. Additionally, for the first time, this study showed that the time between diagnosis and the end of the treatment was significantly shorter with f-URS. The findings of this study should be supported by further prospective randomised studies.
